# Identification of novel blood biomarkers of treatment response in cystic fibrosis pulmonary exacerbations by label-free quantitative proteomics

**DOI:** 10.1038/s41598-019-53759-1

**Published:** 2019-11-20

**Authors:** Kang Dong, Kyung-Mee Moon, Virginia Chen, Raymond Ng, Leonard J. Foster, Scott J. Tebbutt, Bradley S. Quon

**Affiliations:** 1 Centre for Heart Lung Innovation, University of British Columbia, Vancouver, BC Canada; 2 Department of Biochemistry & Molecular Biology, University of British Columbia, Vancouver, Canada; 3 Division of Respiratory Medicine, Department of Medicine, University of British Columbia, Vancouver, BC Canada; 4 PROOF Centre of Excellence, Vancouver, BC Canada; 5 Department of Computer Science, University of British Columbia, Vancouver, BC Canada; 6 Data Science Institute, University of British Columbia, Vancouver, BC Canada

**Keywords:** Predictive markers, Medical research

## Abstract

Pulmonary exacerbations (PEx) are clinically impactful events for individuals with CF. Unfortunately, many CF individuals with PEx fail to regain their baseline lung function despite treatment. The objective of this study was to use unbiased proteomic technology to identify novel blood protein biomarkers that change following intravenous (IV) antibiotic treatment and to explore if changes correlate with clinical response by the end of treatment. Blood samples from 25 PEx events derived from 22 unique CF adults were collected within 24 hours of hospital admission, day 5, day 10, and IV antibiotic completion. Three-hundred and forty-six blood proteins were evaluated with label-free liquid chromatography-tandem mass spectrometry (LC-MS/MS) quantitative proteomics and immunoassays. Forty-seven plasma proteins changed significantly following 5 days of IV antibiotic treatment (q-value ≤ 0.10). Early change in IGF2R from hospital admission to day 5 correlated with overall change in symptom score (CFRSD-CRISS) by the end of treatment (r = −0.48, p-value = 0.04). Several plasma proteins identified and quantified by label-free LC-MS/MS changed early following treatment with IV antibiotics and many of these proteins are involved in complement activation and inflammatory/immune-related pathways. Early change in IGF2R correlated with symptom response following IV antibiotic treatment and requires further validation as a predictive biomarker of symptom response.

## Introduction

Cystic fibrosis (CF) is a life-limiting autosomal recessive disease affecting over 70,000 people worldwide^1^. Individuals with CF experience recurrent pulmonary exacerbations (PEx) that are characterized by intermittent worsening in respiratory signs and symptoms^2–4^ and are associated with increased morbidity and irreversible loss in lung function^5,6^. CF PEx are commonly triggered by respiratory viruses, clonal shifts of colonizing bacteria, and sometimes non-infectious causes (air pollution, medication non-adherence) and are typically treated with antibiotics and increased airway clearance therapies (i.e. hypertonic saline and dornase alfa)^2,4^. Based on data from the 2017 Cystic Fibrosis Foundation Patient Registry, over 40% of CF adults were diagnosed with at least one PEx that required treatment with intravenous (IV) antibiotics^7^. Unfortunately, many individuals with CF fail to regain their baseline lung function despite IV antibiotic treatment^8^.

Suboptimal PEx outcomes may be due to delayed recognition and treatment, widely varied treatment decisions, and differences in the approach to monitoring treatment response, including recovery in lung function and/or resolution in signs and symptoms^9,10^. A novel adjunctive strategy that can provide an additional dimension to the objective monitoring of PEx treatment response is desired as it has the potential to improve clinical outcomes for CF individuals.

Biomarkers that are measured objectively and reproducibly have been studied in order to help guide therapeutic interventions in diseases such as COPD^11–13^. In CF, blood-based biomarkers that reflect systemic inflammation, such as C-reactive protein and calprotectin, decrease significantly following PEx treatment^14–18^. However, for a biomarker to aid in clinical decision making, an early change is potentially more informative in assisting treatment decisions as it provides the opportunity for CF physicians to modify treatments earlier than if they had waited for the patient to respond or not clinically, which can take longer in some patients. Based on the results of a prior study performed by our group, admission but not early change in CRP was found to be useful in predicting treatment outcomes and therefore CRP does not appear to be a useful marker of early response to PEx treatment in CF^19^.

In this study, we recruited adult CF subjects who were diagnosed with a PEx and required hospitalization for IV antibiotic treatment. We prospectively collected blood, symptom diaries, and spirometry (e.g. FEV_1_) within 24 hours of admission, day 5, day 10, and IV antibiotic treatment completion. Blood samples from these CF subjects have previously been evaluated in a prior study using multiple-reaction monitoring mass spectrometry (MRM-MS), a targeted proteomics approach^20^. The objective of this study was to use label-free (untargeted) liquid chromatography-tandem mass spectrometry (LC-MS/MS) to identify novel blood protein biomarkers that are associated with early response to IV antibiotics (i.e. from hospital admission to day 5) and to determine if early changes correlate with clinical outcome by the end of IV antibiotic treatment, in terms of improvement in lung function and symptoms.

## Results

### Clinical characteristics at hospital admission for PEx

A total of 25 PEx events from 22 unique CF subjects were eligible for this study. Clinical characteristics of participating subjects at hospital admission (V1) are summarized in Table 1. In brief, mean baseline lung function was 63.4 (21.5) % predicted and 60% of CF subjects had moderate to severe airflow obstruction (FEV_1 < _70% predicted). Mean FEV_1_% predicted and CF Respiratory Symptom Diary-Chronic Respiratory Infection Symptom Score (CFRSD-CRISS) at V1 were 53.2 (SD 20.7) % predicted and 50.5 (SD 7.5), respectively. The modified Fuchs exacerbation score ranged from 4 to 8 with a median score of 6. Fourteen PEx events (56%) were characterized by a > 10% relative drop in FEV_1_% predicted when compared to baseline. Over half of PEx events (13/25, 52%) were non-acute with the patient describing a change in symptoms for at least two weeks prior to hospitalization and 4 of the PEx events were characterized by the receipt of oral antibiotics prior to hospitalization.

**Table 1:** Clinical characteristics at hospital admission (V1).

Clinical characteristics	
Number of PEx	25
Number of subjects	22
Age, mean (SD)	34.8 (12.9)
Female, No. (%)	12 (48)
Genotype, No. (%)	
ΔF508 Homozygous	11 (50)
$\Delta$F508 Heterozygous	7 (32)
Other (non-$\Delta$F508)	4 (18)
FEV_1_% predicted, mean (SD)	53.2 (20.7)
>10% relative drop FEV_1_% predicted from baseline, No. (%)^a^	14 (56)
BMI, mean (SD)	21.7 (3.5)
CFRSD-CRISS, mean (SD)	50.5 (7.5)
Modified Fuchs Score, median (range)	6 (4 to 8)
Best FEV_1_% predicted in 6 months prior to PEx, mean (SD)	63.4 (21.5)
Best FEV_1_% predicted in 6 months prior to PEx, No. (%)	
<40	4 (16)
40–69	11 (44)
70–89	4 (16)
≥90	6 (24)
Sputum Microbiology, No. (%)	
*P*. *aeruginosa*	14 (56)
MSSA	12 (48)
MRSA	4 (16)
*Burkholderia cepacia complex*	3 (12)
Symptom Onset, No. (%)	
>2 weeks	13 (52)
<2 weeks	12 (48)

Abbreviation: PEx, pulmonary exacerbations; FEV_1_, forced expiratory volume in 1 second; BMI, body mass index; CFRSD-CRISS, CF Respiratory Symptom Diary-Chronic Respiratory Infection Symptom Score; *P*. *aeruginosa*, Pseudomonas aeruginosa; MSSA, Methicillin-sensitive S. aureus; MRSA, Methicillin-resistant S. aureus.

^a^Baseline lung function is defined as the best FEV1% predicted in the 6 months prior to the index PEx.

### Clinical outcomes of PEx treatment

**Figure 1:**
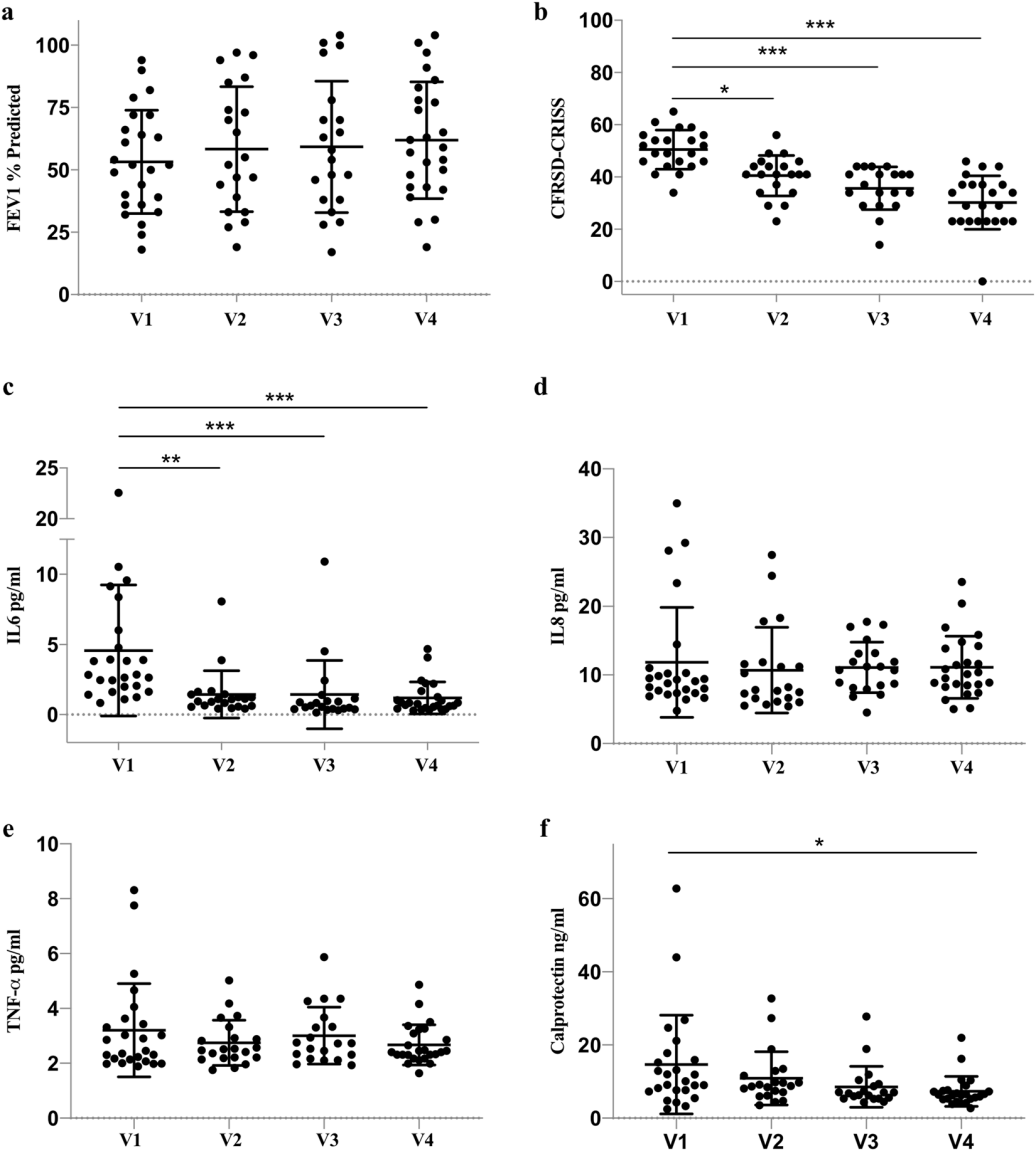
Longitudinal changes in clinical outcomes and candidate blood proteins. Abbreviation: PEx, pulmonary exacerbations; FEV_1_, forced expiratory volume in 1 second; CFRSD-CRISS, CF Respiratory Symptom Diary-Chronic Respiratory Infection Symptom Score; IL-6, Interleukin-6; IL-8, Interleukin-8; TNF-α, Tumor necrosis factor-α; SD, Standard deviation. Statistical significance: p-value < 0.05 (*), p-value < 0.01(**), p-value < 0.001(***).

Clinical outcomes of PEx treatment are summarized in e-Table 1. For 25 PEx events, the median duration of IV antibiotic treatment was 14 days (ranged from 13 to 24 days). The FEV_1_% predicted increased from V1 to each of the subsequent time points (V2, V3, and V4) but overall changes were not significant (e-Table 1, Fig. 1a). Majority of the PEx events (n = 21, 84%) recovered to 90% of baseline lung function but fewer recovered to ≥100% of their baseline lung function (n = 9, 36%). Twenty out of 25 PEx events had symptom questionnaires completed and the mean CFRSD-CRISS decreased significantly from V1 to each of the following time points (V2, V3, V4; e-Table 1, Fig. 1b). Fifteen (60%) events were characterized by >11-point decrease, which has been defined as the minimum clinically important difference^10,21^.

### Correlation between protein levels at hospital admission (V1) with clinical and demographic factors

Serum IL-6 levels at hospital admission (V1) measured with immunoassay inversely correlated with baseline FEV_1_% predicted (r = −0.40, p-value = 0.046; e-Table 2). The levels of 16 plasma proteins measured with LC-MS/MS also significantly correlated with baseline FEV_1_% predicted at V1 (e-Table 2). Moreover, significant correlations were identified between age and many plasma protein abundances at V1 and the correlations varied by sex (e-Table 3).

**Table 2:** Gene ontology (GO) biological process pathway enrichment analysis based on DE proteins from V1 to V2.

GO term ID	Term description	Observed gene count	Background gene count	q-value
GO:0002673	regulation of acute inflammatory response	11	92	2.51E-13
GO:0030449	regulation of complement activation	10	52	2.51E-13
GO:0070613	regulation of protein processing	12	116	2.51E-13
GO:2000257	regulation of protein activation cascade	10	54	2.51E-13
GO:0072376	protein activation cascade	10	74	1.28E-12
GO:0050727	regulation of inflammatory response	13	338	2.60E-10
GO:0002252	immune effector process	18	927	3.73E-10
GO:0006958	complement activation, classical pathway	7	34	9.93E-10
GO:0032101	regulation of response to external stimulus	16	732	1.28E-09
GO:0030162	regulation of proteolysis	16	742	1.43E-09

**Table 3:** Reactome pathway enrichment analysis based on differentially expressed (DE) proteins from V1 to V2.

RCTM term ID	Term description	Observed gene count	Background gene count	q-value
HSA-166658	Complement cascade	11	56	1.21E-15
HSA-977606	Regulation of Complement cascade	10	47	1.10E-14
HSA-168249	Innate Immune System	20	1012	4.56E-12
HSA-168256	Immune System	24	1925	7.71E-11
HSA-109582	Hemostasis	13	601	4.48E-08
HSA-114608	Platelet degranulation	8	125	4.48E-08
HSA-140877	Formation of Fibrin Clot (Clotting Cascade)	6	39	4.48E-08
HSA-76002	Platelet activation, signaling and aggregation	9	256	3.12E-07
HSA-166663	Initial triggering of complement	4	21	8.68E-06
HSA-140837	Intrinsic Pathway of Fibrin Clot Formation	4	22	9.31E-06

### Longitudinal changes in candidate and LC-MS/MS blood proteins

Serum IL-6 levels significantly decreased from hospital admission (V1) to each of the following time points (V2, V3, V4; e-Table 1, Fig. 1c), whereas significant changes were not identified between subsequent time points, which is consistent with IL-6 changing early in response to IV antibiotic therapy. Serum calprotectin levels significantly decreased from hospital admission (V1) to treatment completion (V4) but not at earlier time points (V2, V3). Significant change in the levels of serum IL-8 and TNF-$\alpha$ were not identified between V1 and any of the subsequent time points.

**Figure 2:**
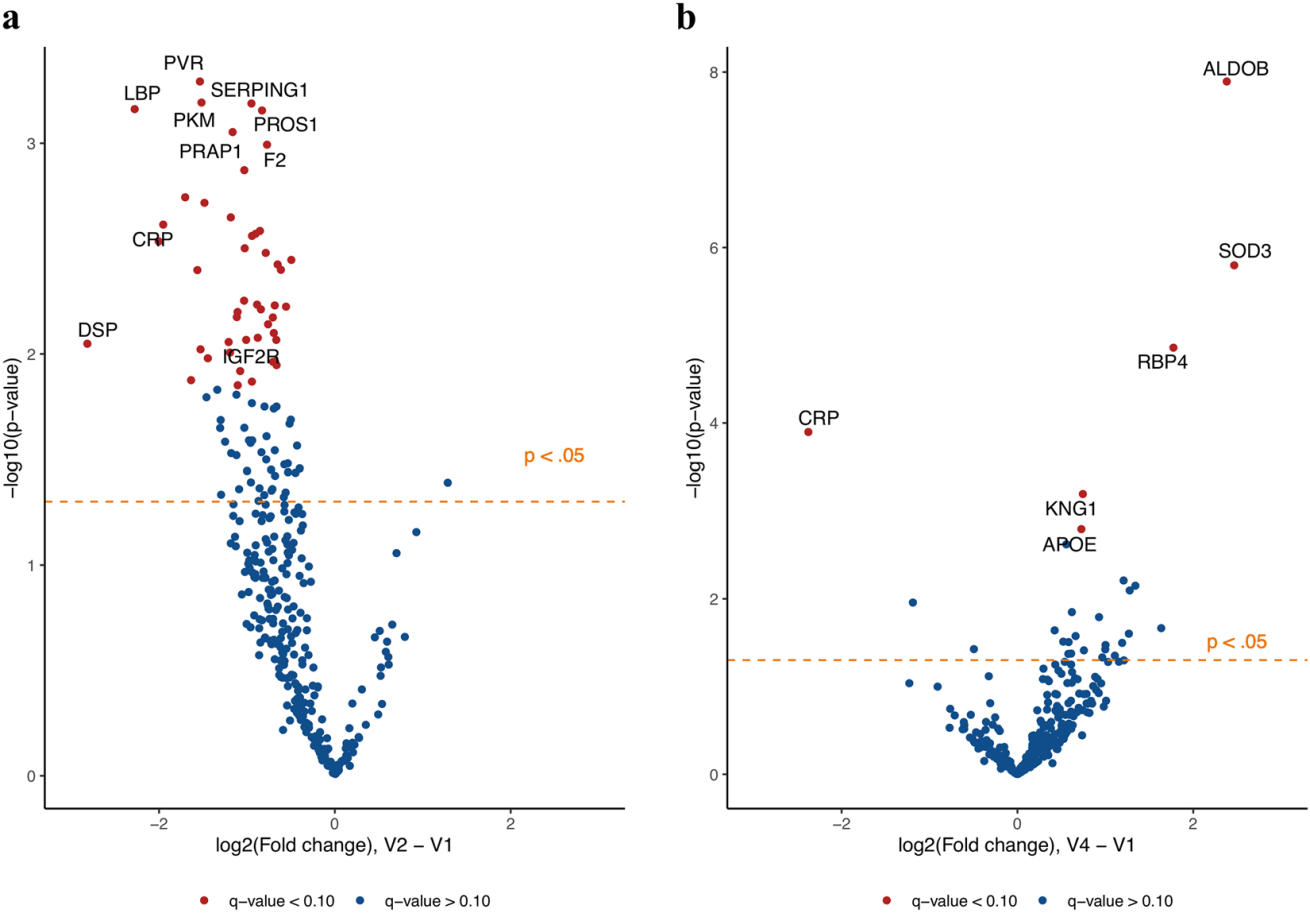
Volcano plot demonstrating blood proteins measured with LC-MS/MS with statistically significant fold-change from: (**a**) V1 to V2 and (**b**) V1 to V4.

Following adjustment for baseline lung function, sex, age, and the interaction between sex and age, 47 proteins changed significantly from V1 to V2 with a FDR cut-off q-value ≤ 0.10 (e-Table 4, Fig. 2a) but just 6 proteins changed significantly from V1 and V4 with a FDR cut-off q-value ≤ 0.10 (e-Table 5, Fig. 2b).

### Protein-protein interaction (PPI) network and pathway enrichment analysis by STRING algorithm

**Figure 3:**
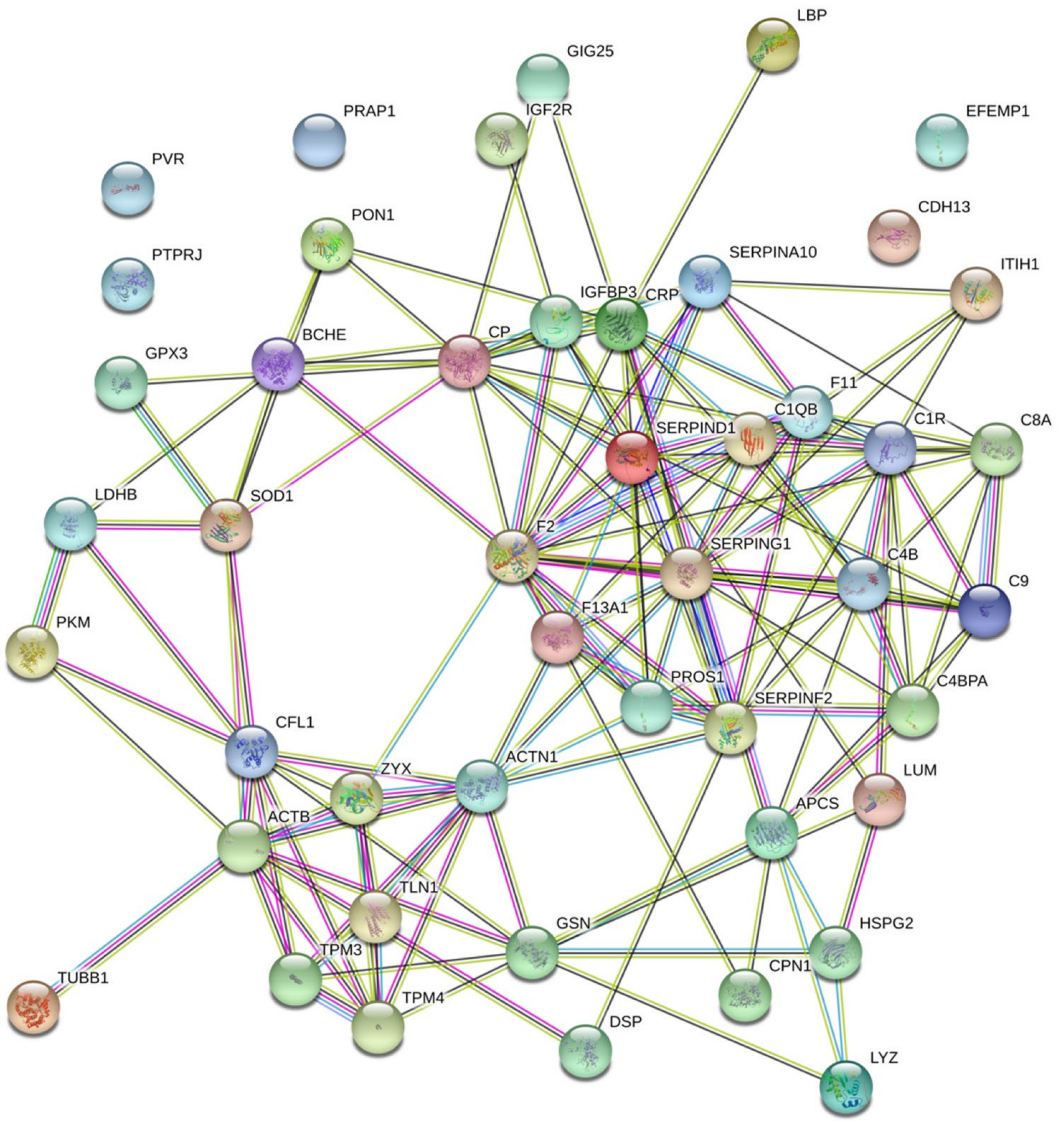
Protein association network based on differentially expressed (DE) proteins from V1 and V2.

The online STRING database (version 11.0) was applied to identify the protein-protein interaction (PPI) network using differentially expressed (DE) proteins and their most enriched molecular pathways following IV antibiotic treatment. The PPI network was constructed by the STRING algorithm after analyzing 47 proteins that changed significantly (q-value ≤ 0.10) between V1 and V2 (Fig. 3) and the top 10 enriched GO biological process terms (Table 2) and Reactome pathways (Table 3) are presented. Twenty-two of the 47 DE proteins from V1 to V2 were involved in the following immune/inflammatory-related GO biological processes: regulation of complement activation, regulation of acute inflammatory response, regulation of inflammatory response, immune effector process, and complement activation/classical pathway (Table 2, e-Fig. 2). Based on the Reactome database, 24 of the 47 DE proteins were involved in 5 immune related pathways (Table 3, e-Fig. 3). Similar analyses were not applied to DE proteins between V1 and V4 since only 6 proteins were identified.

### Correlation between early change in blood protein levels from hospital admission (V1) to Day 5 (V2) with changes in clinical outcomes from hospital admission (V1) to IV antibiotic completion (V4)

Among 47 DE proteins identified and quantified with LC-MS/MS, early change (V1 to V2) in the levels of just one protein, insulin like growth factor 2 receptor (IGF2R), inversely correlated with overall change in CFRSD-CRISS from V1 to V4 (r = −0.48, p-value = 0.04; e-Table 6). No significant correlations were identified between early change in the levels of 47 DE proteins and relative change in FEV_1_% predicted from V1 to V4. Additionally, early change in the levels of candidate blood proteins did not correlate with overall change in either FEV_1_% predicted or CFRSD-CRISS from V1 to V4.

A *post-hoc* sensitivity analysis indicated that the correlation between early change in IGF2R with overall change in CFRSD-CRISS from V1 to V4 was generally consistent when we randomly selected one PEx from each of the three subjects who had repeat PEx (e-Table 6).

### Correlation between overall change in blood protein levels from hospital admission (V1) to IV antibiotic completion (V4) with changes in clinical outcomes from hospital admission (V1) to IV antibiotic completion (V4)

Six plasma proteins identified and measured with LC-MS/MS significantly changed from V1 to V4 but no significant correlations with relative change in FEV_1_% predicted or absolute change in CFRSD-CRISS from V1 to V4 were observed. Similarly, overall change in the levels of candidate blood proteins did not correlate with overall change in either FEV_1_% predicted or CFRSD-CRISS from V1 to V4.

## Discussion

This is the first study to apply untargeted LC-MS/MS quantitative proteomics to identify blood proteins that change in response to IV antibiotics during the treatment of CF PEx. In addition to confirming blood protein biomarkers previously reported to change following IV antibiotics, including serum IL-6, calprotectin and plasma C-reactive protein (CRP), several novel plasma proteins were also identified with LC-MS/MS^16,22^. Interestingly, more proteins exhibited changes early during the treatment course (i.e. by day 5 of treatment) and relatively fewer towards the end of the treatment. Based on pathway enrichment analysis many of these proteins are involved in complement activation and regulation of the inflammatory response/immunity.

Proteins that changed early and remained significant by the end of IV antibiotic treatment included serum IL-6 and plasma CRP. However, early and overall change in serum IL-6 and plasma CRP did not correlate with changes in clinical outcomes, including FEV_1_% predicted and CFRSD-CRISS scores, by the end of treatment. Calprotectin is a candidate marker that also changed significantly by the end of treatment but also did not correlate with changes in clinical outcomes. In contrast, early change (V1 to V2) in insulin like growth factor 2 receptor (IGF2R; also known as cation-independent mannose-6-phosphate receptor or CI-M6PR) levels significantly correlated with symptom response (i.e. CFRSD-CRISS) by the end of IV antibiotic therapy. IGF2R is a multi-functional binding protein capable of binding insulin growth factor 2 (IGF2), mannose-6-phosphate (M6P), and retinoic acid^23^. Depending on the ligand to which it binds, IGF2R is involved in modulating a number of biological pathways including cell migration, wound healing, angiogenesis, apoptosis and the response to viral infection. Although it has not been studied in the context of CF previously, it is also induced by inflammatory mediators and has been studied as a candidate marker for systemic inflammation in other patient populations such as HIV^24^.

Many of the proteins that were found to be downregulated by day 5 of treatment are involved in complement activation (i.e. complement proteins C1r, C1q, C4b, C4b-binding protein, C8, C9). The complement proteins are key components of the innate immune system which promote neutrophilic inflammation and defend against pathogens^25^. Unregulated or persistent complement activation triggers a destructive inflammatory cascade which may lead to lung tissue damage and cause progressive loss of lung function^25,26^. Elevated levels of pro-inflammatory complement proteins have been observed in the sputum of individuals with CF^26^. Despite the potential lung protective effects of downregulating the complement system early during PEx treatment, we did not observe a greater recovery of lung function in such individuals but this study was small and therefore this warrants further study.

This study has a number of important limitations. Only 25 PEx events were analyzed in this exploratory study and therefore this study could have been underpowered to examine relationships between biomarkers and treatment outcomes. Furthermore, there was minimal decrease observed in FEV_1_% predicted at the time of hospitalization in comparison to stable baseline as only half of the PEx events were characterized by a >10% relative drop in FEV_1_% predicted from baseline. As a result, the improvements in FEV_1_% predicted in response to treatment were relatively modest and may have limited the potential to identify significant correlations with changes in blood proteins levels. As this was an untargeted discovery study, a large number of proteins were identified with LC-MS/MS proteomics and found to change significantly following treatment but the multiple statistical comparisons performed could have inflated the type 1 error. As such, the Benjamini-Hochberg method was applied to adjust for multiple-testing with a cut-off q-value of ≤0.10. However, this approach may have been too stringent and resulted in false negatives at this discovery stage.

In conclusion, by using label-free LC-MS/MS quantitative proteomics, we identified several blood proteins involved in complement activation and inflammatory/immune-related pathways that changed in response to IV antibiotic treatment. Early change in IGFR2 correlated with symptom improvement by the end of treatment and requires further validation as an early marker of symptomatic treatment response in individuals with CF.

## Methods

### Study ethics

The research protocol was approved by the University of British Columbia Providence Health Care Research Institute Research Ethics Board (UBC-PHC REB number H12-00835). Informed written consent was obtained from participating subjects. All methods were performed in accordance with the relevant guidelines and regulations to protect human subjects and ensure that participants remain de-identified during samples analysis and data reporting.

### Study cohort

Adult CF subjects were recruited prospectively when diagnosed with a PEx and admitted to St. Paul’s Hospital (Vancouver, BC) for intravenous (IV) antibiotic therapy between July 1, 2013 and June 30, 2015. The PEx events were defined according to changes in respiratory symptoms with a modified Fuchs PEx score of at least 4/12 requiring hospitalization for IV antibiotic treatment^3^. Subjects were excluded if they had a history of solid organ transplantation or were receiving chronic immunosuppressive treatment. Participating subjects received IV antibiotic treatment in conjunction with airway clearance therapies but the IV antibiotic regimen and duration of PEx treatment were left to the discretion of the most responsible CF physician.

### Blood samples and clinical outcomes

Blood samples and clinical outcomes were obtained from subjects within 24 hours of hospitalization for IV antibiotic therapy (V1), treatment day 5 (V2), treatment day 10 (V3), and IV antibiotic treatment completion (V4). Blood samples (serum, EDTA-treated) were collected and processed following standard operating procedures and then stored at −80 °C until thawing for batched analysis.

Clinical characteristics including age, sex, baseline lung function (i.e. FEV_1_% predicted), PEx requiring IV antibiotic treatment in the prior year, sputum microbiology, were collected at hospital admission (V1). Baseline lung function was defined as the best FEV_1_% predicted in the 6 months prior to the index PEx. CF Respiratory Symptom Diary-Chronic Respiratory Infection Symptom Score (CFRSD-CRISS) and FEV_1_% predicted were recorded at V1 and each of the following time points (V2, V3, V4). CFRSD-CRISS is scaled from 0 to 100 points with a higher score indicating more severe respiratory symptoms^21^. Clinical outcomes of interest included absolute change in CFRSD-CRISS and relative change in FEV_1_% predicted from admission (V1) to treatment completion (V4). Absolute change in FEV_1_% predicted was not analyzed as it is largely influenced by baseline lung function with larger increases seen with higher baseline lung function^9,10^.

### Untargeted proteomic profiling of blood proteins

Plasma samples were analyzed with label-free liquid chromatography-tandem mass spectrometry (LC-MS/MS) at the University of British Columbia Proteomics Core Facility. In brief, to facilitate the analysis of less abundant plasma proteins, fourteen of the most highly abundant proteins were first immunodepleted using the Human 14 Multiple Affinity Removal Spin Cartridge (Agilent Technologies, Santa Clara, CA), which removes albumin, immunoglobulin (Ig) G, alpha 1-antitrypsin, IgA, transferrin, haptoglobin, fibrinogen, alpha 2-macroglobulin, alpha 1-acid glycoprotein, IgM, apolipoprotein AI, apolipoprotein AII, complement C3 and transthyretin^27^. Remaining plasma samples were trypsin-digested overnight as previously described^27^. Resulting peptides were desalted and purified with C-18 STop And Go Extraction (STAGE) Tips^28^. Purified peptides were fractionated using the Agilent 1100 HPLC system at 50 μL/min flow rate^29^. The analytical column was operated at 50 °C using an in-house packed 75 μm C18 column heater. The trap column that was added onto the analytical column was a 2 cm-long, 100 μm-inner diameter fused silica, packed with 5 μm-diameter Aqua C-18 beads (Phenomenex, Torrance, CA). Analytical gradient was set at 75 minutes: changing from 10% to 35% Buffer B (80% acetonitrile, 0.1% formic acid) for the first 60 minutes and then wash with 100% Buffer B for 15 minutes. The sample was initially fractionated into 96 wells (45 seconds per well) then pooled in a noncontiguous manner (every 6th well was pooled) resulting into 6 fractions for further LC-MS/MS analysis^29^. Six fractions were then loaded into the Impact II Q-ToF mass spectrometer (Bruker, Germany)^30^. Peptides identified by LC-MS/MS were searched with MaxQuant software (version 1.5.3.30) with default label-free-quantitation setting and match-between runs options enabled^31^. All plasma samples were evaluated in duplicate and mean values were used for analyses. The LC-MS/MS data were deposited in the PRoteomics IDEntifications (PRIDE) database under accession number PXD016089.

### Analysis of candidate blood proteins

Five low-abundance candidate blood proteins, including interleukin (IL)-${\rm{\beta }}$, IL-6, IL-8, tumor necrosis factor (TNF)-$\alpha$, and calprotectin, that are beyond the detection limits of LC-MS/MS were analyzed in serum samples with multiplex electrochemiluminescence immunoassays (Meso Scale Discovery, Carlsbad, CA). Among these five low-abundance blood proteins, IL-1 ${\rm{\beta }}$ was below the detection limits for most of the samples, and therefore, was excluded from subsequent analyses. All assays were performed in duplicate with mean coefficient of variation (CV) < 5% and mean values were used for analyses.

### Protein-protein interaction (PPI) network and pathway enrichment analysis

The differentially expressed (DE) proteins identified by LC-MS/MS were applied as inputs for PPI network and pathway enrichment analysis. The STRING database (version 11.0) was utilized to assess the protein functional association^32^. The active protein-protein interactions were identified based on experimentally determined interactions and curated databases, such as Gene ontology, KEGG, Reactome databases. The predicted protein-protein interactions included gene neighborhood, gene fusion, gene co-occurrence, text-mining, co-expression, and protein homology^32^. Enriched Gene Ontology (GO) biological process term and Reactome pathways were reported and proteins involved in the immune and inflammation related pathways were highlighted in the constructed PPI network.

### Statistical analyses

Statistical analyses were performed using R (version 3.5.0, the R Foundation for Statistical Computing, Vienna, Austria) and Prism 8 (GraphPad, La Jolla, CA). Continuous variables were presented as mean ± standard deviation (SD). Categorical variables were reported as number with proportions. Longitudinal changes in the levels of candidate blood proteins and changes in clinical outcomes (i.e., FEV_1_% predicted and CFRSD-CRISS) were analyzed with non-parametric Kruskal-Wallis test followed by the post-hoc Dunn’s test to correct for multiple comparisons.

LC-MS/MS data was pre-processed as described in the flow diagram (e-Fig. 1). Proteins were excluded if they were identified as reversed and/or contaminated by MaxQuant software during peptide searching or detected in less than 25% of blood samples. Three-hundred and forty-one proteins passed the quality control matrix. Missing values from these 341 proteins were imputed with half of the minimum abundance of each protein across the analyzed samples and then, the levels of proteins were log2 transformed before subsequent analyses. Fold-changes of blood proteins from V1 to V2 and V1 to V4 measured with LC-MS/MS were analyzed with the limma R software package and adjusted for baseline lung function, sex, age, and an interaction term between sex and age. The Benjamini-Hochberg method was applied to correct for multiple comparisons and false discovery rate (FDR) adjusted p-values (q-value) ≤ 0.10 were reported for differentially expressed (DE) proteins.

To assess how blood protein levels at V1 might be confounded by baseline disease severity and demographic factors, correlation between blood protein levels at V1 and age, sex, and baseline lung function were evaluated with Spearman’s correlation. Additionally, correlations between early change (V1 to V2) and overall change (V1 to V4) in blood proteins with relative change in FEV_1_% predicted and absolute change in CFRSD-CRISS from V1 to V4 were examined with Spearman’s correlation. Statistical significance was reported when two-sided p-values were ≤0.05.

To ensure our findings were robust, we performed a *post-hoc* sensitivity analysis that randomly selected one PEx from three subjects who had repeat PEx.

## Supplementary information

Dataset 3

Supplementary materials

Dataset 1

Dataset 2

## Data Availability

The datasets generated during the current study are available in the PRoteomics IDEntifications (PRIDE) database under accession number PXD016089. Protein quantification results from MaxQuant software (version 1.5.3.30), study cohort characteristics, and R scripts used for statistical analyses are included in the Supplementary materials files.
