# Supplementary material for: Identification of novel blood biomarkers of treatment response in cystic fibrosis pulmonary exacerbations by label-free quantitative proteomics
Source: Sci Rep. 2019 Nov 20;9:17126. doi: 10.1038/s41598-019-53759-1 (PMC6868239; doi:10.1038/s41598-019-53759-1)
Supplement: Supplementary file 2 — Supplementary materials [file 41598_2019_53759_MOESM2_ESM.pdf]

Supplemental Materials

Identification of novel blood biomarkers of treatment response in cystic fibrosis pulmonary exacerbations by label-free quantitative proteomics

Kang Dong[1], Kyung-Mee Moon[2], Virginia Chen[1, 4], Raymond Ng[4, 5, 6], Leonard J. Foster[2], Scott J. Tebbutt[1, 3, 4], Bradley S. Quon[1, 3] *

1. Centre for Heart Lung Innovation, University of British Columbia, Vancouver, BC, Canada
2. Department of Biochemistry & Molecular Biology, University of British Columbia, Vancouver, Canada
3. Division of Respiratory Medicine, Department of Medicine, University of British Columbia, Vancouver, BC, Canada
4. PROOF Centre of Excellence, Vancouver, BC, Canada
5. Department of Computer Science, University of British Columbia, Vancouver, BC, Canada
6. Data Science Institute, University of British Columbia, Vancouver, BC, Canada

e-Table 1. Longitudinal changes in FEV$_1$% predicted, CFRSD-CRISS, and candidate blood proteins with PEx treatment.

Clinical Outcomes	Hospital admission (V1, n = 25)	Day 5 (V2, n = 21)	Day 10 (V3, n = 20)	Treatment completion (V4, n = 25)
**Days on IV antibiotics, median (range)**	**--**	**--**	**--**	**14 (13 to 24)**
**Recovered ≥ 90% of baseline FEV$_1$% predicted, No. (%)**	**--**	**--**	**--**	**21 (84)**
**FEV$_1$% predicted, No.**	**25**	**20**	**20**	**25**
**mean (SD)**	**53.2 (20.7)**	**58.3 (25.0)**	**59.3 (26.3)**	**61.9 (23.4)**
**Relative change from V1, mean (SD)**	**--**	**10% (20%)**	**20% (20%)**	**18% (20%)**
**CFRSD-CRISS decrease > 11 points, No. (%)**	**--**	**--**	**--**	**15 (60)**
**CFRSD-CRISS, No.**	**22**	**20**	**20**	**23**
**mean (SD)**	**50.5 (7.5)**	**40.5 (7.8)**	**35.7 (8.2)**	**30.3 (10.2)**
**Absolute change from V1, mean (SD)**	**--**	**-11.1 (8.3)***	**-16.5 (9.4)*****	**-21.4 (12.7)*****
**Candidate blood proteins (No.)**	**25**	**21**	**20**	**25**
**IL-6 (pg/ml), mean (SD)**	**4.6 (4.7)**	**1.4 (1.7)**	**1.4 (2.4)**	**1.2 (1.1)**
**Absolute change from V1, mean (SD)**	**--**	**-3.4 (4.0)****	**-3.5 (3.4)*****	**-3.4 (4.2)*****
**IL-8 (pg/ml), mean (SD)**	**11.8 (8.0)**	**10.7 (6.3)**	**11.1 (3.7)**	**11.1 (4.5)**
**Absolute change from V1, mean (SD)**	**--**	**-1.7 (6.5)**	**-1.4 (7.0)**	**-0.7 (6.5)**
**TNF-α (pg/ml), mean (SD)**	**3.2 (1.7)**	**2.7 (0.8)**	**3.0 (1.0)**	**2.7 (0.7)**
**Absolute change from V1, mean (SD)**	**--**	**-0.6 (1.6)**	**-0.4 (1.5)**	**-0.5 (1.6)**
**Calprotectin (ng/ml), mean (SD)**	**14.7 (13.5)**	**10.9 (7.3)**	**8.6 (5.6)**	**7.3 (4.1)**
**Absolute change from V1, mean (SD)**	**--**	**-4.9 (9.8)**	**-7.9 (12.1)**	**-7.3 (12.2)***

Abbreviation: PEx, pulmonary exacerbations; FEV$_1$, forced expiratory volume in 1 second; CFRSD-CRISS,CF Respiratory Symptom Diary-Chronic Respiratory Infection Symptom Score; IL-6, Interleukin-6; IL-8, Interleukin-8; TNF-α, Tumor necrosis factor-α; SD, Standard deviation. **Statistical significance**: p-value < 0.05 (*), p-value < 0.01(**), p-value < 0.001(***).

e-Table 2. Protein levels at V1 significantly correlated with baseline FEV_1% predicted

Protein	Gene	Rho	p-value
**MSD**			
Interleukin-6	IL-6	-0.403	0.046
Tumor necrosis factor-α	TNF-α	-0.279	0.176
Interleukin-8	IL-8	0.050	0.814
Calprotectin	S100A8/9	-0.041	0.846
**LC-MS/MS**			
Neural cell adhesion molecule L1-like protein	CHL1	-0.625	0.001
Mannose-binding protein C	MBL2	-0.609	0.001
Basement membrane-specific heparan sulfate proteoglycan core protein	HSPG2	-0.592	0.002
Tyrosine-protein phosphatase non-receptor type 18	PTPN18	-0.545	0.005
Ig gamma-1 chain C region	IGHG1	-0.532	0.006
Ig alpha-1 chain C region	IGHA1	-0.504	0.010
Myosin-9	MYH9	0.502	0.011
Glucosidase 2 subunit beta	PRKCSH	0.472	0.017
Cadherin-6	CDH6	-0.442	0.027
Platelet basic protein	PPBP	-0.438	0.028
Nucleoside diphosphate kinase	NME1-NME2	-0.431	0.031
Fructose-bisphosphate aldolase A	ALDOA	-0.431	0.032
Corticosteroid-binding globulin	SERPINA6	-0.418	0.038
C-reactive protein	CRP	-0.407	0.043
Glutathione peroxidase 3	GPX3	-0.401	0.047
Apolipoprotein C-I	APOC1	0.399	0.048

e-Table 3. Blood protein levels at V1 significantly correlated with age.

		All Subjects		Male Subjects Only		Female Subjects Only	
**Protein**	**Gene**	**Rho**	**p-value**	**Rho**	**p-value**	**Rho**	**p-value**
**Voltage-dependent calcium channel subunit alpha-2/delta-1**	**CACNA2D1**	-0.55	**0.004**	-0.63	0.021	-0.52	0.085
**Plasminogen**	**PLG**	0.44	**0.029**	0.74	0.004	0.11	0.728
**Beta-Ala-His dipeptidase**	**CNDP1**	0.44	**0.027**	0.31	0.297	0.46	0.133
**Collectin-11**	**COLEC11**	-0.44	**0.028**	-0.51	0.076	-0.36	0.248
**Cartilage acidic protein 1**	**CRTAC1**	0.41	**0.039**	0.44	0.135	0.61	0.034
**Protein AMBP**	**AMBP**	0.27	0.190	0.58	**0.039**	0.08	0.795
**Pantetheinase**	**VNN1**	0.38	0.063	0.75	**0.003**	0.31	0.328
**Complement C1r subcomponent**	**C1R**	0.28	0.174	0.59	**0.034**	-0.08	0.812
**Fibronectin**	**FN1**	-0.06	0.761	0.56	**0.046**	-0.48	0.117
**Coagulation factor XI**	**F11**	0.26	0.205	0.66	**0.014**	0.09	0.778
**C4b-binding protein alpha chain**	**C4BPA**	0.37	0.070	0.75	**0.003**	-0.05	0.871
**Plasma serine protease inhibitor**	**SERPINA5**	0.32	0.119	0.67	**0.012**	0.12	0.712
**Alpha-actinin-1**	**ACTN1**	0.16	0.436	0.63	**0.021**	-0.18	0.570
**Heat shock 70 kDa protein 6**	**HSPA6**	0.17	0.428	0.56	**0.047**	-0.33	0.289
**Tenascin-X**	**TNXB**	-0.12	0.572	0.26	0.386	-0.60	**0.041**
**Insulin-like growth factor-binding protein 3**	**IGFBP3**	-0.21	0.313	0.21	0.493	-0.59	**0.044**
**Alpha-2-macroglobulin**	**A2M**	-0.38	0.062	0.01	0.964	-0.78	**0.003**
**L-lactate dehydrogenase A chain**	**LDHA**	-0.36	0.077	-0.18	0.547	-0.58	**0.048**
**Ig gamma-1 chain C region**	**IGHG1**	-0.30	0.150	-0.01	0.972	-0.69	**0.013**
**Ig alpha-1 chain C region**	**IGHA1**	-0.31	0.127	0.04	0.901	-0.58	**0.048**
**Apolipoprotein(a)**	**LPA**	0.39	0.053	0.29	0.334	0.58	**0.049**
**Gamma-enolase**	**ENO2**	-0.20	0.334	0.28	0.352	-0.70	**0.012**
**Protein deglycase DJ-1**	**PARK7**	-0.25	0.229	-0.02	0.936	-0.61	**0.035**
**Calmodulin-like protein 5**	**CALML5**	-0.37	0.072	-0.16	0.603	-0.67	**0.016**

e-Table 4. Blood proteins with statistically significant fold-change in levels from V1 to V2 measured with LC-MS/MS

Protein	Gene	Fold-Change mean	Direction V1 to V2	p-value	q-value
Poliovirus receptor	PVR	0.35	Down	0.001	0.048
Pyruvate kinase PKM	PKM	0.35	Down	0.001	0.048
Plasma protease C1 inhibitor	SERPING1	0.52	Down	0.001	0.048
Lipopolysaccharide-binding protein	LBP	0.21	Down	0.001	0.048
Vitamin K-dependent protein S	PROS1	0.56	Down	0.001	0.048
Proline-rich acidic protein 1	PRAP1	0.45	Down	0.001	0.049
Prothrombin	F2	0.59	Down	0.001	0.049
Coagulation factor XIII A chain	F13A1	0.49	Down	0.001	0.057
Glutathione peroxidase 3	GPX3	0.31	Down	0.002	0.059
Receptor-type tyrosine-protein phosphatase eta	PTPRJ	0.36	Down	0.002	0.059
Cofilin-1	CFL1	0.44	Down	0.002	0.059
C-reactive protein	CRP	0.26	Down	0.002	0.059
Serum amyloid P-component	APCS	0.55	Down	0.003	0.059
Complement C1r subcomponent	C1R	0.54	Down	0.003	0.059
Complement component C9	C9	0.52	Down	0.003	0.059
Alpha-actinin-1	ACTN1	0.25	Down	0.003	0.059
Superoxide dismutase [Cu-Zn]	SOD1	0.49	Down	0.003	0.059
L-lactate dehydrogenase B chain	LDHB	0.58	Down	0.003	0.059
Gelsolin	GSN	0.71	Down	0.004	0.059
Complement component C8 alpha chain	C8A	0.64	Down	0.004	0.059
Inter-alpha-trypsin inhibitor heavy chain H1	ITIH1	0.65	Down	0.004	0.059
Basement membrane-specific heparan sulfate proteoglycan core protein	HSPG2	0.34	Down	0.004	0.059
Cholinesterase	BCHE	0.49	Down	0.006	0.074
Protein Z-dependent protease inhibitor	SERPINA10	0.54	Down	0.006	0.074
Heparin cofactor 2	SERPIND1	0.62	Down	0.006	0.074
Carboxypeptidase N catalytic chain	CPN1	0.68	Down	0.006	0.074
Alpha-1-antichymotrypsin	SERPINA3	0.56	Down	0.006	0.074
Tropomyosin alpha-3 chain	TPM3	0.46	Down	0.006	0.074
Serum paraoxonase/arylesterase 1	PON1	0.46	Down	0.007	0.074
Insulin-like growth factor-binding protein 3	IGFBP3	0.61	Down	0.007	0.074
Lumican	LUM	0.59	Down	0.007	0.077
Complement C1q subcomponent subunit B	C1QB	0.62	Down	0.008	0.080
Actin, cytoplasmic 1	ACTB	0.54	Down	0.008	0.080
Coagulation factor XI	F11	0.50	Down	0.009	0.080
Complement C4-B	C4B	0.63	Down	0.009	0.080
Zyxin	ZYX	0.43	Down	0.009	0.080
Desmoplakin	DSP	0.14	Down	0.009	0.080

e-Table 4. (cont'd). Blood proteins with statistically significant fold-change in levels from V1 to V2 measured with LC-MS/MS

Protein	Gene	Fold-Change mean	Direction V1 to V2	p-value	q-value
C4b-binding protein alpha chain	C4BPA	0.35	Down	0.010	0.083
Talin-1	TLN1	0.44	Down	0.010	0.084
EGF-containing fibulin-like extracellular matrix protein 1	EFEMP1	0.37	Down	0.010	0.086
Cadherin-13	CDH13	0.62	Down	0.011	0.086
Alpha-2-antiplasmin	SERPINF2	0.61	Down	0.011	0.086
Ceruloplasmin	CP	0.63	Down	0.011	0.088
Insulin like growth factor 2 receptor	IGF2R	0.47	Down	0.012	0.091
Tubulin beta-1 chain	TUBB1	0.32	Down	0.013	0.098
Lysozyme C	LYZ	0.52	Down	0.014	0.098
Tropomyosin alpha-4 chain	TPM4	0.46	Down	0.014	0.100

e-Table 5. Blood proteins with statistically significant fold-change in levels from V1 to V4 measured with LC-MS/MS

Protein	Gene	Fold-change mean	Direction V1 to V4	p-value	q-value
Fructose-bisphosphate aldolase B	ALDOB	5.21	Up	<0.001	< 0.001
Extracellular superoxide dismutase [Cu-Zn]	SOD3	5.52	Up	<0.001	< 0.001
Retinol-binding protein 4	RBP4	3.42	Up	<0.001	0.002
C-reactive protein	CRP	0.19	Down	<0.001	0.011
Kininogen-1	KNG1	1.68	Up	0.001	0.044
Apolipoprotein E	APOE	1.66	Up	0.002	0.091

e-Table 6. Blood proteins with early change in levels from V1 to V2 and that correlate with changes in CFRSD-CRISS from V1 to V4 in comparison to candidate proteins measured with MSD.

Protein	Gene	Rho	p-value
**LC-MS/MS**			
Insulin like growth factor 2 receptor	IGF2R	-0.48	0.04
***post-hoc* sensitivity analysis**			
Insulin like growth factor 2 receptor [a], mean (SD)	IGF2R	-0.49 (0.05)	0.09 (0.08)
**MSD**			
Interleukin-6	IL-6	0.20	0.43
Tumor necrosis factor-α	TNF-α	-0.29	0.24
Interleukin-8	IL-8	0.02	0.94
Calprotectin	S100A8/9	0.04	0.86

[a] Randomly selected one PEx from three subjects who had repeat PEx. Mean (SD) was calculated based on 6 repeat exams.

e-Figure legends

e-Figure 1. Flow diagram of LC-MS/MS data pre-processing.

e-Figure 2. GO biological process pathway analysis of DE Proteins from V1 toV2.
Color-coded nodes: regulation of complement activation (red); regulation of acute inflammatory response (purple); regulation of inflammatory response (green); immune effector process (yellow); complement activation, classical pathway (pink).

e-Figure 3. Reactome pathway analysis of DE Proteins from V1 toV2.
Color-coded nodes: complement cascade (red); regulation of complement cascade (purple); innate immune system (green); immune system (yellow); initial triggering of complement (pink).

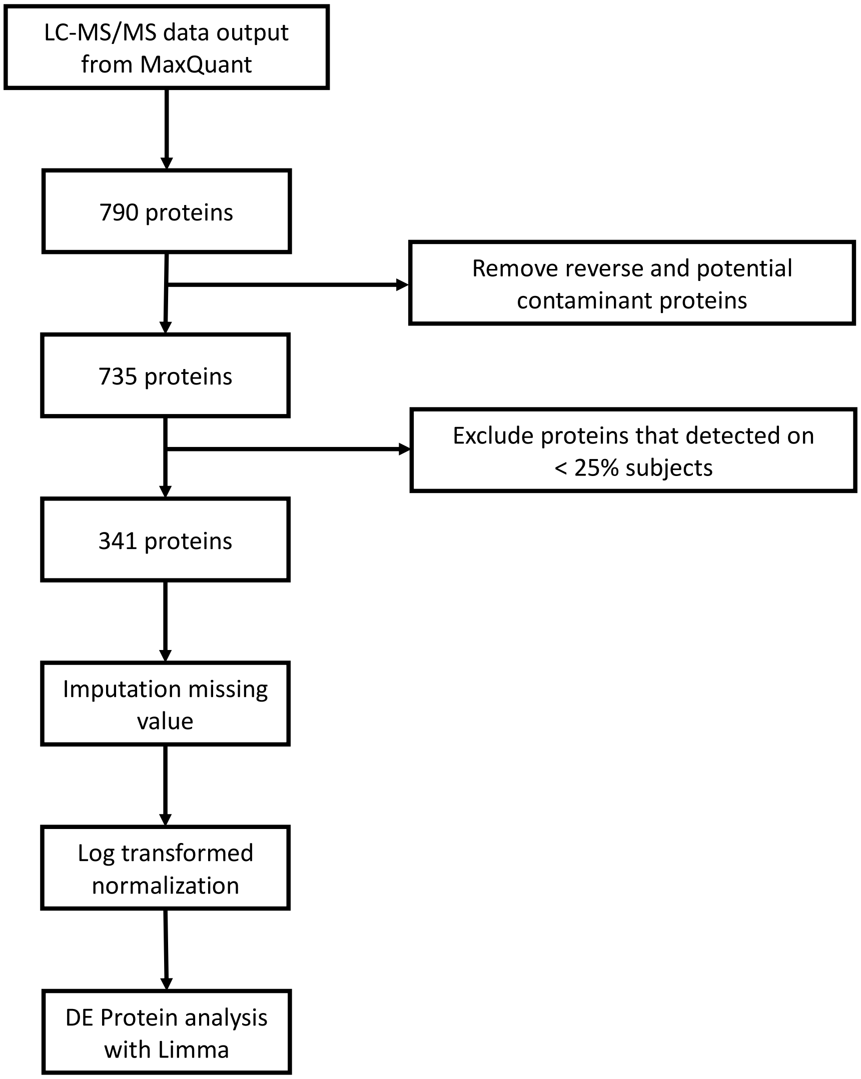
LC-MS/MS data output from MaxQuant
790 proteins
Remove reverse and potential contaminant proteins
735 proteins
Exclude proteins that detected on < 25% subjects
341 proteins
Imputation missing value
Log transformed normalization
DE Protein analysis with Limma

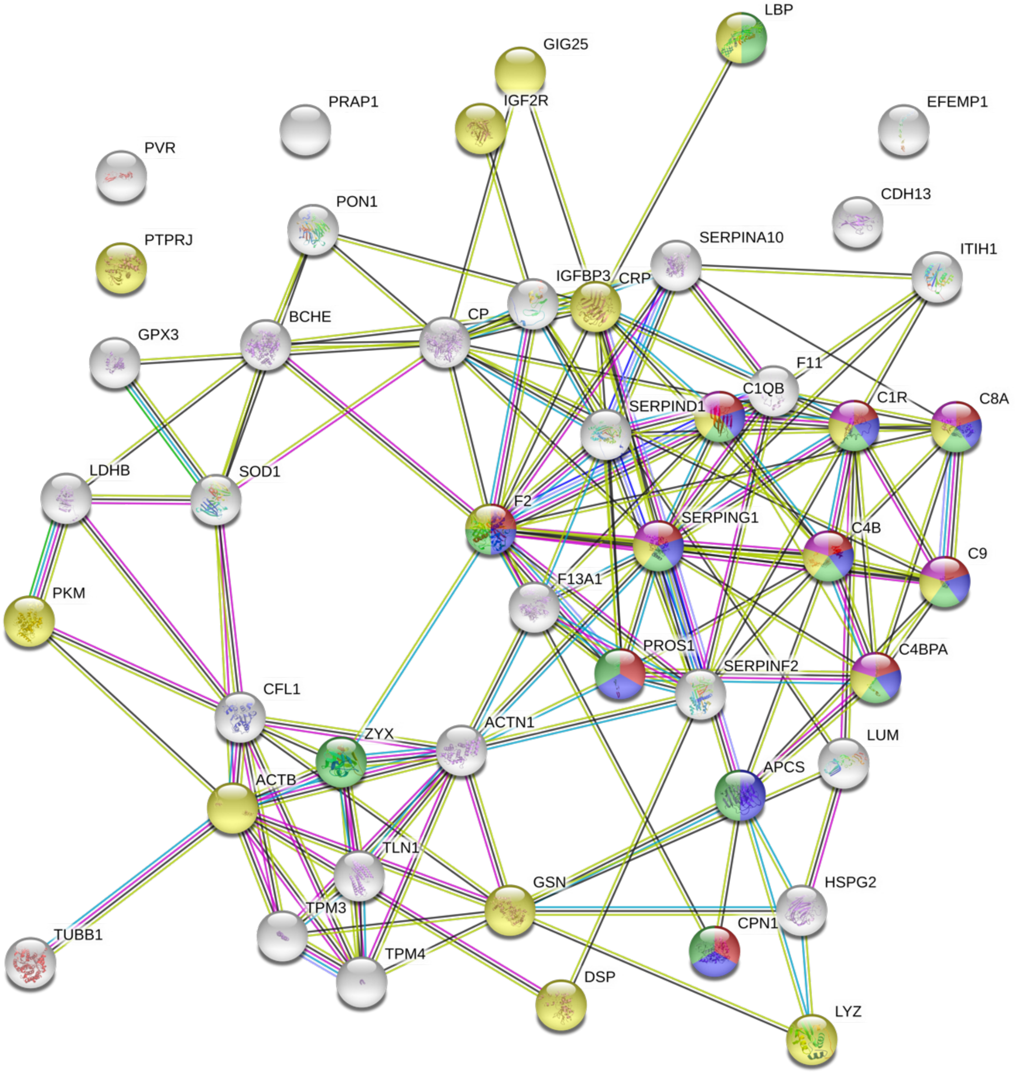
LBP
GIG25
IGF2R
PRAP1
EFEMP1
PVR
PON1
CDH13
PTPRJ
SERPINA10
ITIH1
IGFBP3
CRP
GPX3
BCHE
CP
F11
C1QB
C1R
C8A
SERPIND1
LDHB
SOD1
F2
SERPING1
C4B
C9
F13A1
PKM
PROS1
SERPINF2
C4BPA
CFL1
ZYX
ACTN1
LUM
ACTB
APCS
TLN1
GSN
HSPG2
TPM3
CPN1
TUBB1
TPM4
DSP
LYZ

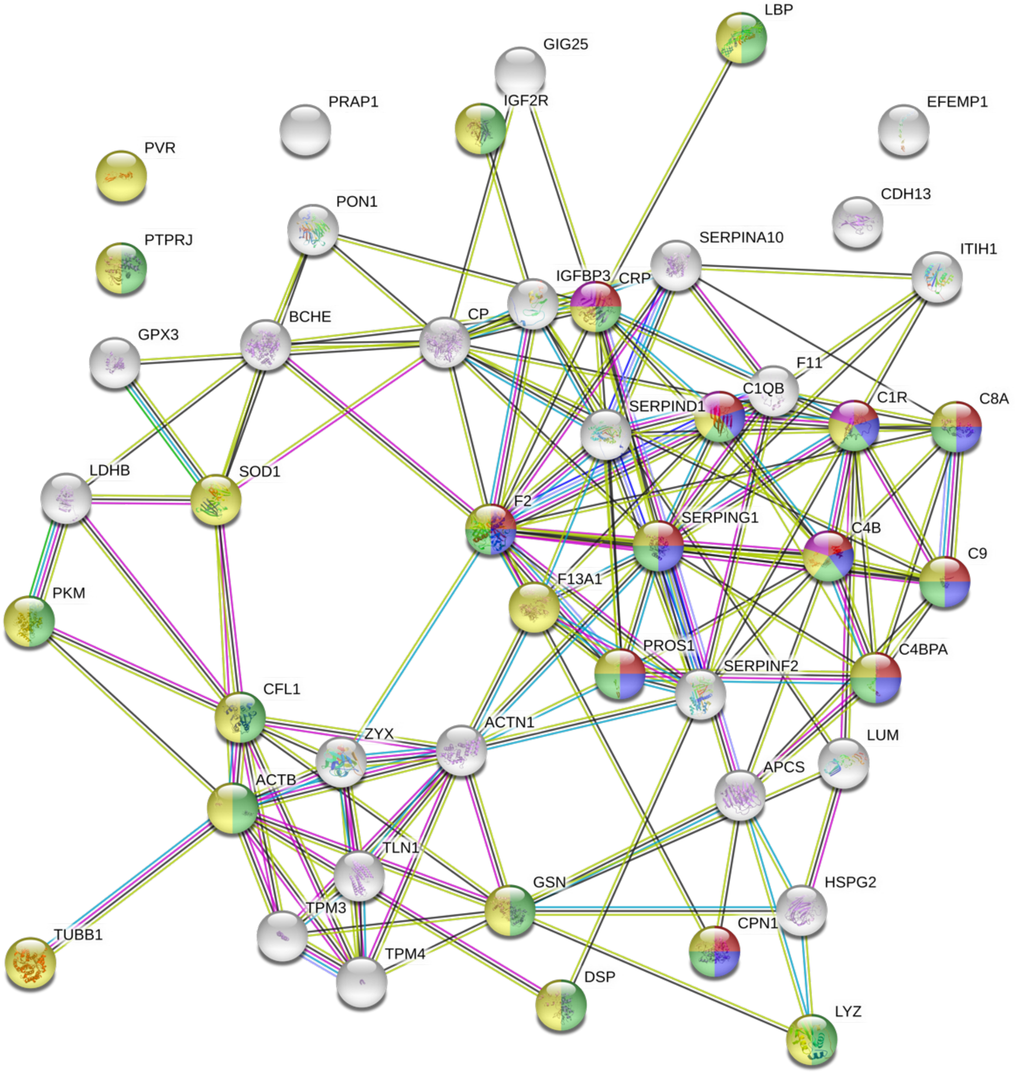

LBP
GIG25
PRAP1
IGF2R
EFEMP1
PVR
CDH13
PON1
SERPINA10
PTPRJ
ITIH1
IGFBP3
CRP
BCHE
CP
GPX3
F11
C1QB
SERPIND1
C1R
C8A
LDHB
SOD1
F2
SERPING1
C4B
C9
F13A1
PKM
PROS1
SERPINF2
C4BPA
CFL1
LUM
ZYX
ACTN1
APCS
ACTB
TLN1
HSPG2
GSN
TPM3
CPN1
TUBB1
TPM4
DSP
LYZ
